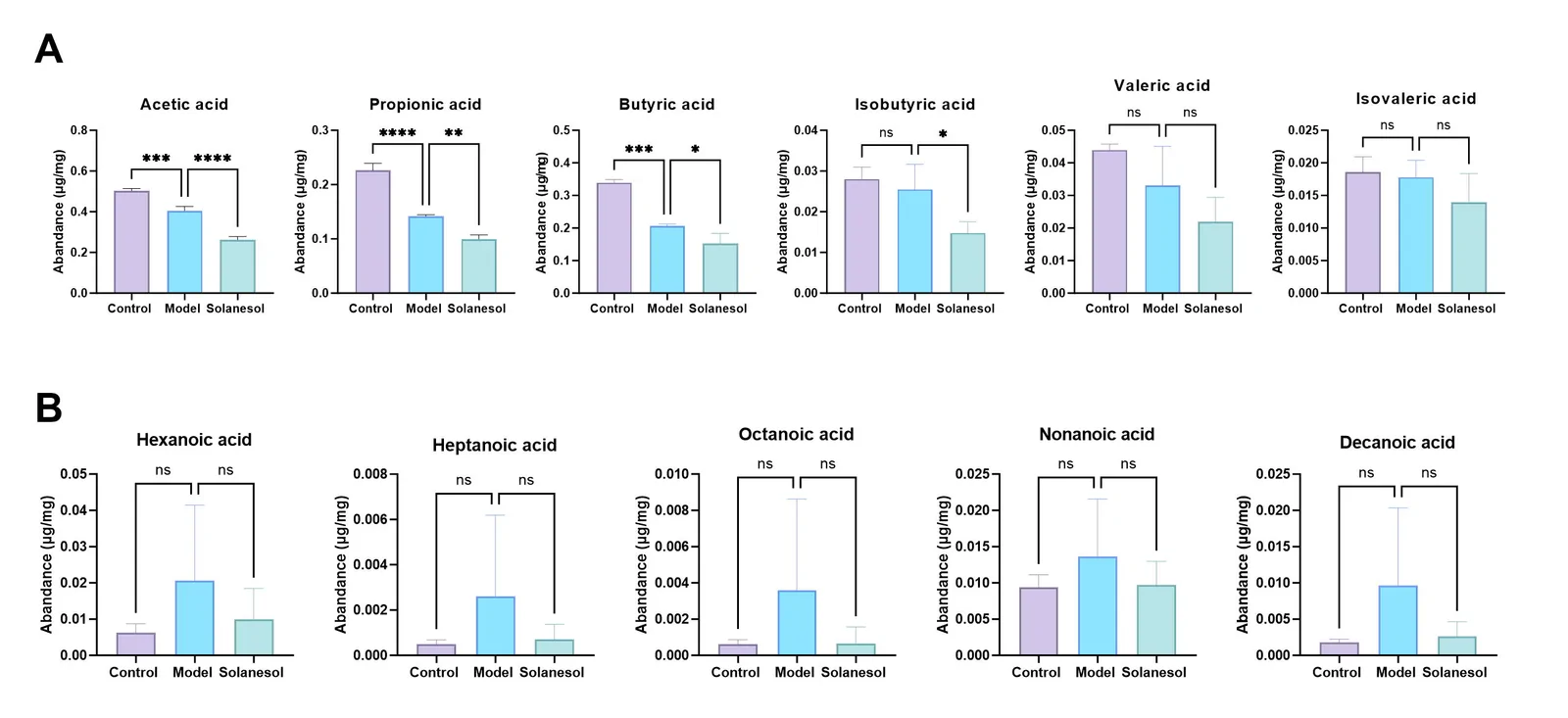# Integrated multi-omics unravels solanesol’s multi-organ protection mechanisms against insulin resistance in type 2 diabetic mice

**DOI:** 10.1016/j.jare.2025.12.025

**Published:** 2025-12-26

**Authors:** Wenji Zhang, Wenli Cheng, Jiaqi Fu, Ruidong Zeng, Zhangying Wang, Jingbo Zhou, Yibo Chen, Wenjuan Zhang

**Affiliations:** a Key Laboratory of Crop Genetic Improvement of Guangdong Province, Crops Research Institute, Guangdong Academy of Agricultural Sciences, Guangzhou 510640, China; b Department of Public Health and Preventive Medicine, School of Medicine, Jinan University, Guangzhou, Guangdong 510632, China; c Rice Research Institute, Guangdong Academy of Agricultural Sciences / Guangdong Key Laboratory of New Technology in Rice Breeding / Guangdong Laboratory for Lingnan Modern Agriculture, Guangzhou 510640, China

**Keywords:** Solanesol, Type 2 diabetes mellitus, Lipid/redox homeostasis, Gut microbiota-metabolite

## Abstract

**Introduction:**

Integrated multi-omics analysis has revolutionized the investigation of plant-derived compounds for type 2 diabetes mellitus (T2DM). Solanesol, a bioactive constituent from Solanaceae plants, exhibits high oral bioavailability and translational potential for multi-target therapeutics.

**Objectives:**

This study aimed to elucidate the multi-target mechanisms and multi-organ protective effects of solanesol in T2DM management through integrated multi-omics approaches, to bridge the gap between phytochemical discovery and clinical translation.

**Methods:**

In Lepr^db/db^ murine models (8–13 weeks), solanesol (1–15 mg/kg/day, i.g.) was compared with metformin (200 mg/kg/day). ​​Serum biochemical parameters were quantified using ELISA/colorimetric assays. Quantitative pathology analysis was performed on H&E/PAS/Masson-stained sections for assessment of hepatic steatosis and glycogenesis. Mitochondrial respiratory chain complexes (I-V) expression and activities were validated by qRT-PCR and ELISA assay, respectively. Multi-omics profiling included gut microbiota sequencing (16S rRNA), fecal metabolomics/lipidomics (UHPLC-Q LC-MS/MS), and hepatic transcriptome/proteome analysis (RNA-seq, 4-Dimensional Label-Free Quantification (4D-LFQ)). Long-/medium-/short-chain fatty acids were detected by GC–MS.

**Results:**

Solanesol improved glucose tolerance, insulin sensitivity, and reduced serum lipids, hepatic gluconeogenesis, uric acid, white adipose mass, pancreatic/hepatic inflammation, and renal fibrosis. Mechanistically, solanesol: 1) enriched beneficial gut microbiota (*Alistipes*, *Anaerotruncus*, and *Parasutterella*) and increased levels of long-chain unsaturated fatty acids; 2) rebalanced the dysfunctional mitochondrial oxidative phosphorylation​​ microenvironment by modulating the expression and the activities of respiratory chain Complexes I-V; 3) modulated hepatic lipid metabolism by ​inhibiting​​ *de novo* ​​lipogenesis​​ via the Acly-Acaca-Fasn pathway, promoting cholesterol efflux and fatty acid oxidation​​ through Abca1/Fabp5, and attenuating inflammation​​ via Lpl-PPARδ downregulation.

**Conclusion:**

Solanesol demonstrates multi-organ protective effects through gut microbiota-metabolite crosstalk and hepatic lipid/redox homeostasis regulation. Its multi-target efficacy and oral bioavailability position it as a novel, clinically translatable candidate for T2DM management.

## Highlights

• Solanesol alleviates type 2 diabetes symptoms and complications.
• It reduces blood glucose, lipid, and uric acid levels effectively.
• Anti-inflammatory effects are shown in liver, pancreas, and kidneys.
• Liver protection is achieved by reducing mitochondrial oxidative stress.
• It modulates lipid metabolism and improves the gut microenvironment.

## Graphical abstract

**Figure:**
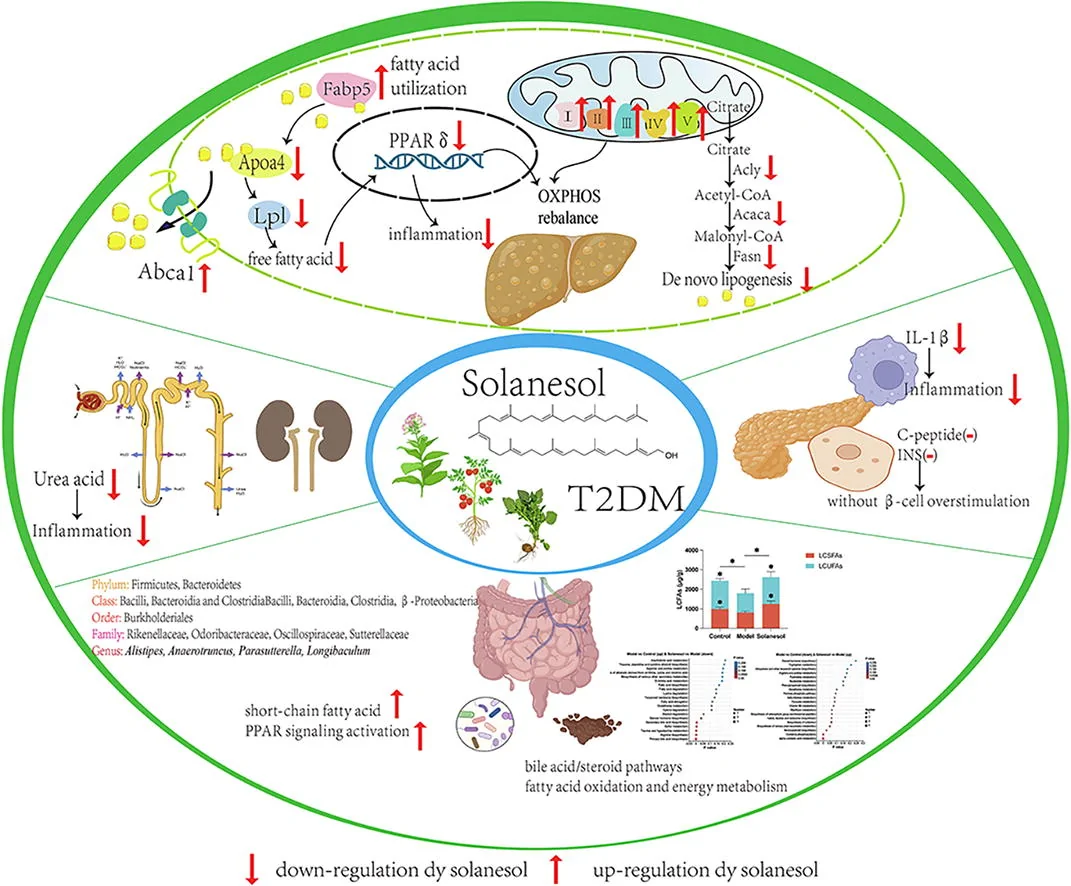

## Introduction

Diabetes mellitus (DM), a multifactorial metabolic disorder driven by genetic-environmental interplay, has reached pandemic proportions globally [1], [2]. In 2021, 10.5 % of adults aged 20–79 (536.6 million) were affected, with projections surging to 12.2 % (783.2 million) by 2045. China is one of the fastest growing countries in the world, and also the country with the largest number of diabetes patients, with 140 million diabetes patients in 2021, an increase of about 71.5 % in a decade [3]. Type 2 diabetes (T2DM), constituting 90–95 % of cases, triggers systemic vascular complications-cardiovascular mortality, renal failure, retinopathy, and neuropathy-imposing a projected $1 trillion healthcare burden by 2045 [4]. The disease's complexity arises from multi-organ dysregulation involving hepatic gluconeogenesis, pancreatic β-cell dysfunction, intestinal microbiota imbalance, and skeletal muscle insulin resistance [5], necessitating holistic therapeutic strategies targeting these interconnected pathways.

Natural products have reemerged as promising candidates for multi-target diabetes management. Over 1/3 of the 1,881 new drugs of FDA-approved drugs (1981–2019) originate from natural scaffolds, including metformin-derived from *Galega officinalis* guanidine compounds. Ginsenosides, betulinic acid, and asiaticoside demonstrate glycemic control via AMPK activation and oxidative stress mitigation due to their structural specificity and relatively little toxicity [6], [7], [8]. Solanesol (C_45_H_74_O), a tetrasesquiterpenoid with nine *trans*-conjugated double bonds, exhibits lipid antioxidant properties and strong free radical scavenging capacity. It is abundant in tobacco, potatoes, tomatoes and other solanesol plant stems and leaves, with its content in tobacco reaching up to 4 % of the dry weight [9], [10]. It serves as a key intermediate in the synthesis of ubiquinone compounds including coenzyme Q10, vitamin K2, and the anticancer synergist N-solanesyl-N [11]; however, studies focusing on its direct pharmacological activities have been limited. A body of research has documented its neuroprotective effects in animal models of cerebral hemorrhage, autism spectrum disorder, Huntington's disease, and bipolar disorder models-characterized by mitochondrial functional restoration [12], [13], [14], [15]. Relevant to the present study, the reports [16], [17] show that solanesol has detoxification effects on alcohol-induced liver injury cells and high-sugar induced liver cell models, mainly by improving oxidative stress balance and mitochondrial function. However, prior *in vitro* studies using contaminated LO2 hepatocytes (HeLa-admixed) necessitate rigorous *in vivo* validation of its mechanisms [18]. A recent study further demonstrated that solanesol alleviates NLRP3 inflammasome activation and macrophage inflammation in adipose tissue in vivo [19]. Nevertheless, no evidence has been reported on its efficacy in treating T2DM, highlighting both the novelty and significance of our current study.

Our study addresses these gaps through comprehensive pharmacodynamic evaluation in Lepr^db/db^ mice, a progressive T2DM model. We demonstrate that solanesol (5 mg/kg/day, 8–13 weeks, i.g.) achieves glycemic control comparable to metformin (200 mg/kg/day) in improving insulin resistance (HOMA-IR: 84.8 % vs 81.8 %), while conferring multi-organ benefits. Comprehensive multi-omics analyses and histopathological evaluations demonstrate that solanesol exerts multi-organ therapeutic effects in T2DM by resolving systemic inflammation, restoring mitochondrial homeostasis, reprogramming lipid metabolism, and remodeling gut microbial ecology, positioning solanesol as a novel multi-target therapeutic agent for T2DM and its comorbidities. These findings identify solanesol as a promising natural lead compound for developing next-generation phytopharmaceuticals targeting T2DM.

## Materials and methods

### Animals and treatment

Six C57BL/6J and thirty db/db mice (specific pathogen-free, male, 7 weeks old) (Beijing, SCXK 2019–0008) were purchased from Beijing Huafukang Biotechnology Co., Ltd. (Beijing, China). These mice were housed in a 12/12-h light and dark cycle at a constant temperature (25 °C ± 2°C) and provided with a normal diet and free water. After 7 days of adaptive feeding, six C57BL/6J mice were divided into the control group and thirty db/db mice were randomly divided into 5 groups (Model group, Metformin group, Solanesol-L group, Solanesol-M group, Solanesol-H group) according to body weight (n = 6 per group). Carboxymethyl cellulose (CMC) is an anionic water-soluble biopolymer that showed promising characteristics as drug delivery, especially in oral [20]. The control and model groups were administered with 0.5 % CMC-Na. The Metformin group was administrated with 0.5 % CMC-Na and 200 mg/kg metformin. The Solanesol-L/M/H group was given 0.5 % CMC-Na and different concentrations of Solanesol (1 mg/kg, 5 mg/kg, 15 mg/kg), respectively. All mice were orally gavaged once daily for 45 days. Glucose tolerance test (GTT) was performed at the end of 3 and 5 weeks. Insulin tolerance test (ITT) was performed at the end of 4 and 6 weeks. The weights of the mice were recorded daily. Food and water intake were recorded weekly on a per-cage basis (with six mice per cage), excluding the two-day periods immediately before and after the GTT and ITT. Fecal samples were collected within 24 h before the end of the experiment. After all experiments, the mice were fasted for 12 h and then euthanized for blood and other tissue collection. The whole blood samples were from *retro*-orbital blood collection and then separated by centrifugation to obtain serum. Liver, kidney, white fat (inguinal white adipose tissue, epididymal white adipose tissue, retroperitoneal white adipose tissue) and brown fat (interscapular brown adipose tissue) were separated and weighed. A part of the liver and left kidney was stored in 4 % paraformaldehyde for subsequent stain. The rest of the samples were stored at −80 °C for subsequent biochemical experiments.

### GTT & ITT

After fasting for 16 h, 2 g/kg 50 % glucose was injected intraperitoneally to test glucose tolerance. After fasting for 6 h, human insulin 0.75 U/kg was injected intraperitoneally to test insulin tolerance. Blood glucose levels were measured before, 30 min, 60 min and 120 min after injection. Blood glucose was detected by Sinocare GA-3 blood glucose meter in mice from the tail vein. The area under the curve (AUC) was calculated by blood glucose and injection time.

### Biochemical analysis

The biochemical indicators in serum were measured using reagent kits of total cholesterol (TC) (A111-1–1), triglyceride (TG) (A110-1–1), high-density lipoprotein cholesterol (HDL-C) (A112-1–1), low-density lipoprotein cholesterol (LDL-C) (A113-1–1), non-esterified fatty acids (NEFA) (A042-2–1), alanine aminotransferase (ALT) (C009-2–1), aspartate aminotransferase (AST) (C010- 2–1), albumin (ALB) (A028-2–1), uric acid (UA) (C012-2–1) and urea assay kit (BUN) (C013-2–1) from Nanjing Jiancheng Bioengineering Institute Co., Ltd. (Nanjing, China). The TC and TG levels in the liver were detected using the same reagent kit as serum. The mouse glycosylated serum protein (GSP) (MM-44373 M1) and mouse insulin (INS) (MM-0579 M1) ELISA Kit from Jiangsu Meimian Industrial Co., Ltd. (Jiangsu, China).

### Histopathological analysis

The fixed liver and kidney tissues were embedded in paraffin. Sections with a thickness of 5 μm were obtained and stained using a hematoxylin and eosin (H&E) staining kit (C0105S) from Shanghai Beyotime Biotechnology Co., Ltd. (Shanghai, China) as well as a periodic acid schiff (PAS) stain kit (G1280) and a modified Masson's trichrome stain kit (G1346) from Beijing Solarbio Science & Technology Co., Ltd. (Beijing, China). All sections were observed under a motorized multifunctional upright fluorescence microscope (DM6000B, Leica, Germany). The proportion of stain area was measured by ImageJ2 (version 2.9.0). The histologic features were assessed with the NASH clinical research network scoring system[21]. The NAFLD activity score (NAS) was calculated as the unweighted sum of the scores for steatosis (0–3), lobular inflammation (0–3), and ballooning (0–2).

### 16S rRNA sequencing of fecal

Total microbial genomic DNA was extracted from fecal samples using the E.Z.N.A.® soil DNA Kit (Omega Bio-tek, USA). The quality and concentration of DNA were determined by agarose gel electrophoresis and a NanoDrop2000 spectrophotometer (Thermo Scientific, USA). The hypervariable region V3-V4 of the bacterial 16S rRNA gene was amplified with primer pairs 338F (5′-ACTCCTACGGGAGGCAGCAG-3′) and 806R (5′-GGACTACHVGGGTWTCTAAT-3′) by PCR System (GeneAmp 9700, Applied Biosystems, USA). The sample using FastPfu DNA Polymerase (AP221-02, TransGen Biotech, Beijing, China) was amplified in a reaction volume of 20 μL including 4 μL 5 × FastPfu buffer, 2 μL 2.5 mM dNTPs, 0.8 μL each primer (5 μM), 0.4 μL Fast Pfu polymerase, 0.2 μL BSA, 10 ng of template DNA and ddH2O. Amplicons were pooled in equimolar amounts and paired-end sequenced on an Illumina Novaseq PE300 sequencing platform (Illumina, San Diego, USA) performed at Shanghai Majorbio Bio-pharm Biotechnology Co., Ltd. (Shanghai, China). Raw data was quality-filtered by fastp (Version 0.19.6) and merged by FLASH (Version 1.2.11). PICRUSt predicted the functional composition of microbial communities. Wilcoxon’s test was used for each group compared with the model group in α-diversity indexes as well as abundances of species and metabolites.

### Non-targeted metabolomics of fecal

A 50 mg fecal sample mixed with 400 μL of extraction reagent (methanol: water = 4:1, v/v) containing 0.02 mg/mL internal standard (L-2-chlorophenylalanine), ground for 6 min (−10 °C, 50 Hz), low-temperature ultrasonic waved for 30 min with (5 °C, 40 kHz), left for 30 min (−20 °C) and followed by centrifuging at 4 °C for 15 min (4 °C, 13000g). The supernatant was transferred to the injection vial for LC-MS/MS analysis. The LC-MS/MS analysis was conducted on a Thermo UHPLC-Q Exactive HF-X system equipped with an ACQUITY UPLC HSS T3 column (100 mm × 2.1 mm i.d., 1.8 μm; Waters, USA). The mobile phases consisted of 0.1 % formic acid in water: acetonitrile (95:5, v/v) (solvent A) and 0.1 % formic acid in acetonitrile: isopropanol: water (47.5:47.5:5, v/v/v) (solvent B). The loading quantity of samples was 3 μL and the column temperature was 40℃. The pretreatment of LC/MS raw data was performed by Progenesis QI software (Waters, USA). Internal standard peaks and any known false positive peaks were removed from the data matrix. Compounds were mainly identified by databases, HMDB (https://www.hmdb.ca/) and Metlin (https://metlin.scripps.edu/).

### Fatty acid analysis

Long-chain fatty acids (LCFAs)​​ were analyzed as follows. A standard mixture of 51 fatty acids was serially diluted in n-hexane to generate a calibration curve (1–2000 μg/mL). Fecal samples were homogenized in chloroform–methanol (2:1) with glass beads, sonicated, and centrifuged. The supernatant was derivatized with 1 % H_2_SO_4_ in methanol at 80 °C for 30  min. After cooling, fatty acid methyl esters were extracted with n-hexane, washed with ice-cold water, and dried over anhydrous Na_2_SO_4_. The extract was spiked with methyl salicylate (internal standard) and analyzed by GC–MS (Thermo Trace 1300/ISQ 7000) using a TG-FAME column and the following temperature program: 80 °C (1  min), to 160 °C at 20 °C/min (1.5  min), to 196 °C at 3 °C/min (8.5  min), to 250 °C at 20 °C/min (3  min). Helium flow was 0.63  mL/min; injector, ion source, and transfer line temperatures were 250 °C, 300 °C, and 280 °C, respectively. EI ionization was used at 70  eV in SIM mode.

Short-chain fatty acids (SCFAs)​​ and medium-chain fatty acid (MCFA) were extracted from feces with water by ball milling and ultrasonication on ice. After centrifugation, the supernatant was acidified with 50 % H_2_SO_4_ and extracted with MTBE containing an internal standard. The organic phase was analyzed using a SHIMADZU GC2030-QP2020 NX gas chromatography-mass spectrometer equipped with a HP-FFAP column. Injection was in split mode (5:1) at 220 °C; helium flow was 1.2  mL/min. The oven program was: 50 °C (1  min), to 150 °C at 50 °C/min (1  min), to 170 °C at 10 °C/min, to 225 °C at 25 °C/min (1  min), to 240 °C at 40 °C/min (1  min). The transfer line, quad, and ion source temperatures were 240 °C, 150 °C, and 240 °C, respectively. EI-MS detection was performed at 70  eV in Scan/SIM mode (*m*/*z* 33–150) with the *m*/*z* range of 33–150 after a solvent delay of 3.75 min.

### Mitochondrial respiratory chain complexes (complex I-IV) activity assay

This study measured mitochondrial complex I–IV levels in liver tissue using commercial ELISA kits (MM-44745 M1, MM-44757 M1, MM-44750 M1, MM-47091 M1, Jiangsu Meimian Industrial Co., Ltd.). Tissue samples were homogenized in cold PBS with protease inhibitors (1:9, w/v) and centrifuged at 5000 × g for 10 min at 4 °C. The supernatant was diluted 5-fold for analysis. Standards or samples (50 μL) were added to the plate along with HRP-conjugated detection antibody (100 μL) and incubated at 37 °C for 60 min. After washing, color was developed using substrates A and B (37 °C, 15 min), stopped with stop solution, and OD450 was measured. Concentrations were determined from standard curves and multiplied by the dilution factor.

### Transcriptome sequencing

Total RNA was extracted from the hepatic tissue using MJZol total RNA extraction kit (Majorbio, China). Then RNA quality was quantified and determined by NanoDrop2000 spectrophotometer (Thermo Scientific, USA) and 5300 Bioanalyser (Agilent Technologies). The RNA sequencing is based on the Illumina Novaseq 6000 sequencing platform. RNA purification, reverse transcription, library construction and sequencing were performed at Shanghai Majorbio Bio-pharm Biotechnology Co., Ltd. (Shanghai, China) according to the manufacturer’s instructions (Illumina, San Diego, CA). The quality check of RNA sequencing was carried out using MultiQC [22]. RNA reads were aligned to mouse reference genome using HISAT2 [23] followed by counting RNA reads by featureCounts [24], one of programs in Subread package. Genes with zero read counts for all samples were removed. In terms of bioanalysis, PCoA was based on the unweighted Unifrac distance. Pareto was used to scale the data before PLS-DA. PLS-DA model-derived variable important in projection (VIP) scores > 1.0 and P < 0.05 in Wilcoxon’s test were used as criteria for differentially expressed metabolites. P < 0.05 was used to define differentially expressed genes (DEGs) in DESeq2 software. The KEGG database was used for pathway enrichment analysis.

### Proteomic profiling

Liver proteomic analyses were performed by Biotree Biotech Co., Ltd using state-of-the-art 4D label-free quantification (4D-LFQ) workflows. Tissue proteins were extracted with urea lysis buffer and quantified via BCA assay. Sequential reduction and alkylation were performed with 5 mM dithiothreitol (30 min, 55°C) and 15 mM iodoacetamide (30 min, dark), respectively. Proteins were digested with trypsin (1:100 w/w, 37 °C, 16 h) and then remove sodium deoxycholate. Peptides were desalted and purified using C18 solid-phase extraction cartridges (Waters).

Approximately 200 ng of peptides were separated on a nanoElute2 UPLC system (Bruker) using a 75 μm × 15 cm PePSep C18 column (1.9 μm particles, Bruker) at 300 nL/min. The 60-min gradient comprised: 2 % B (0–0 min), 5–22 % B (0–45 min), 22–37 % B (45–50 min), 37–80 % B (50–55 min), and 80 % B (55–60 min), where mobile phase A was 0.1 % formic acid/water and B was 0.1 % formic acid/acetonitrile. MS data were acquired on a timsTOF Pro2 mass spectrometer (Bruker) operated in DDA-PASEF mode (*m*/*z* 100–1700). PASEF MS/MS scans utilized collision energy ramping from 20 eV (1/K0 = 0.6 Vs/cm^2^) to 59 eV (1/K0 = 1.6 Vs/cm^2^), optimizing fragmentation efficiency across ion mobility ranges.

Proteomic data were performed using protti packages for data processing, statistical analysis, and abundance calculation [25]. To ensure robustness in downstream analyses, missingness was categorized using the assign_missingness function in protti, which distinguishes between missing at random (MAR) and missing not at random (MNAR) based on predefined completeness thresholds. Missing values were imputed using the impute function in protti. Imputed log2-transformed intensities were then subjected to differential abundance analysis using a two-sample *t*-test via the calculate_diff_abundance function. Proteins were classified as significantly differentially abundant based on a log2 fold change (log2FC) threshold of 1 and a p-value cutoff of 0.05. Pathway enrichment analysis was performed by Metascape [26], results were filtered by absolute p-value < 0.05, and FDR value < 0.05. Gene Set Enrichment Analysis (GSEA) was performed by GSEA software with threshold of NES value > 1, p-value < 0.05, and FDR value < 0.05 [27].

### qRT-PCR

Hepatic RNA extraction was performed using TRIzol reagent (Invitrogen) with standardized protocols. Briefly, 30–50 mg frozen liver tissues were homogenized in 1 mL TRIzol, followed by chloroform phase separation and isopropanol precipitation. RNA pellets were washed with 75 % ethanol and dissolved in RNase-free water. RNA integrity was verified by agarose gel electrophoresis (RIN > 8.0) and quantified using NanoDrop 2000 (Thermo Fisher).

For cDNA synthesis, 1 μg total RNA was reverse-transcribed using PrimeScript RT Master Mix (Takara) in a 20 μL reaction (37 °C/15 min, 85 °C/5 s). cDNA was stored at −80 °C until use. Primers for target genes and reference gene Actb were designed via PrimerBank and validated using NCBI Primer-BLAST, with sequences synthesized by IGE Biotechnology (Guangzhou).

*Ndufa5* F: ATGGCGGGCTTGCTGAAAA; R: GCTGCATGTTTAGGAAAGTGCTT;

*Cox6b1* F: ACTACCTGGACTTCCACCG; R: ACCCATGACACGGGACAGA.

*Ndufb4* F: CTTGATTCGCTGGACCTATGC; R: GGAGTGGGCCTGAAATTAGGA.

*Uqcrb* F: GGCCGATCTGCTGTTTCAG; R: CATCTCGCATTAACCCCAGTT.

*Atp5h* F: GCTGGGCGTAAACTTGCTCTA; R: CAGACAGACTAGCCAACCTGG.

*Actb* F: GGCTGTATTCCCCTCCATCG; R: CCAGTTGGTAACAATGCCATGT).

Acly F: AGGAAGTGCCACCTCCAACAGT; R: CGCTCATCACAGATGCTGGTCA.

*Acaca* F: GTTCTGTTGGACAACGCCTTCAC; R: GGAGTCACAGAAGCAGCCCATT.

*Abca1* F: GGAGCCTTTGTGGAACTCTTCC; R: CGCTCTCTTCAGCCACTTTGAG.

*Fabp5* F: GACGACTGTGTTCTCTTGTAACC; R: TGTTATCGTGCTCTCCTTCCCG.

*Apoa4* F: CAGAAGACGGATGTCACTCAGC; R: AGCTGTACGACAAAGGGCACCA.

*Fasn* F: CACAGTGCTCAAAGGACATGCC; R: CACCAGGTGTAGTGCCTTCCTC.

*PPARδ* F: GGACCAGAACACACGCTTCCTT; R: CCGACATTCCATGTTGAGGCTG.

*Lpl* F: GCGTAGCAGGAAGTCTGACCAA; R: AGCGTCATCAGGAGAAAGGCGA.

Quantitative PCR was conducted using TB Green Premix Ex Taq II (Takara) on a CFX96 system (Bio-Rad). Each 25 μL reaction contained 0.4 μM primers and < 100 ng cDNA template. Thermal cycling included: initial denaturation at 95°C/30 s, 40 cycles of 95 °C/5 s and 60°C/30 s, followed by melt curve analysis (65-95°C, 0.5°C increments). Gene expression was normalized to Actb and calculated using the 2^−ΔΔCt^ method.

### Statistical analyses

Phenotypic characteristics were statistically analyzed and visualization by GraphPad Prism 9 (Version 9.4.0). One-way ANOVA and Dunnett’s test were used for comparisons among groups and pairwise in weight change, feed intake, water intake, AUC of GTT and ITT, organ coefficient, serum and liver biochemical indices, and PAS staining proportion. Kruskal-Wallis test and Dunnett’s test were used for comparisons among groups and pairwise in NAS. All data are presented as the means ± SD. *P* < 0.05 was considered as significant.

## Results

### Solanesol ameliorates diabetic metabolic symptoms

**Fig. 1:**
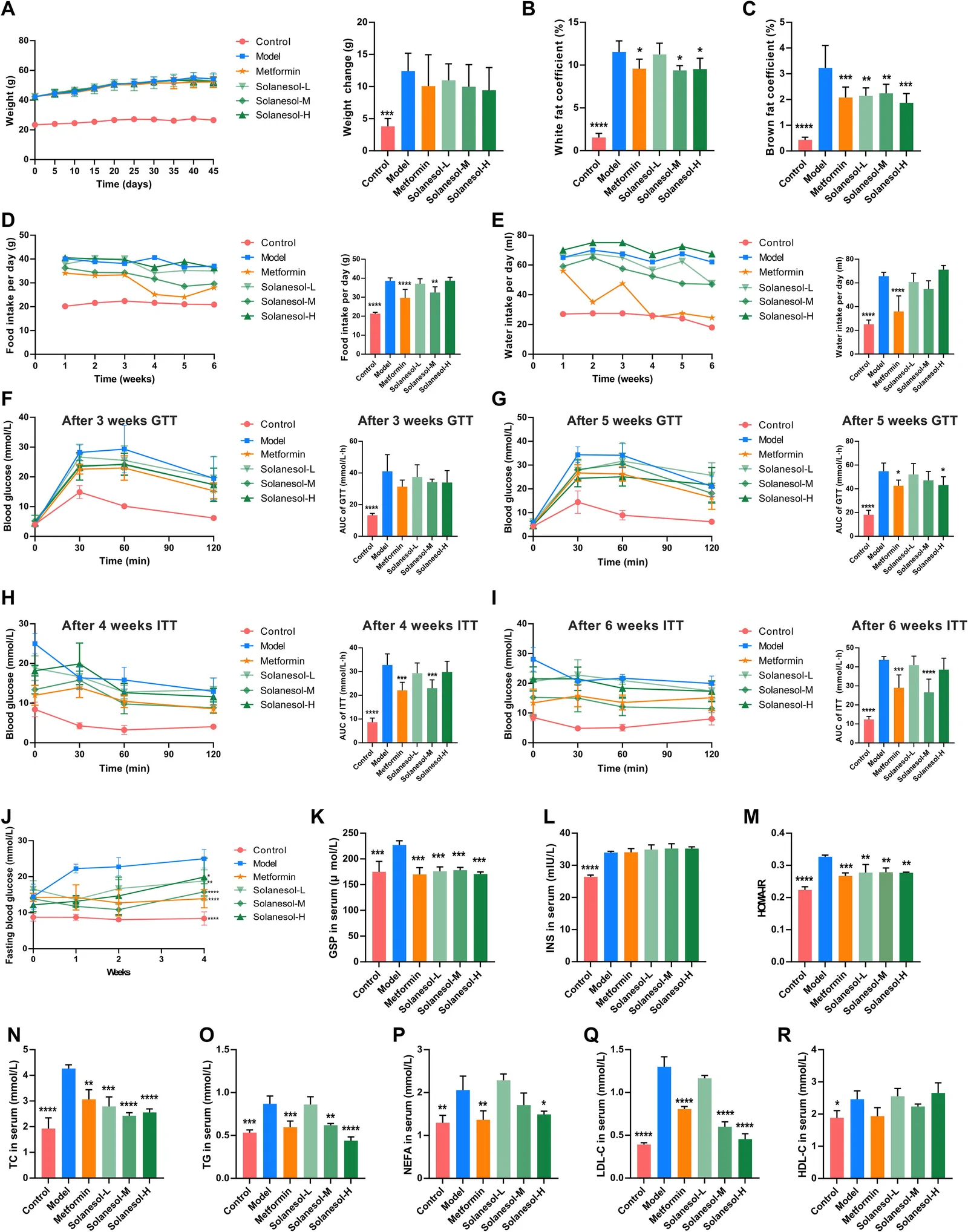
Solanesol improves metabolic parameters in obese T2DM mice. (A) Body weight and body weight change. (B) White adipose tissue coefficient. (WAT) weight coefficient. (C) Brown fat coefficient. (D) Food intake. (E) Water consumption. (F) Glucose tolerance test (GTT) after 3 weeks treatment. (G) Glucose tolerance test (GTT) after 5 weeks. (H) Insulin tolerance test (ITT) after 4 weeks treatment. (I) Insulin tolerance test (ITT) after 6 weeks treatment. (J) Fasting blood glucose (FBG) levels during the first four weeks. (K-M) Serum glucose index (GSP, INS, HOMA-IR). (N-R) Serum lipid parameter (TC, TG, NEFA, LDL-c, HDL-c). Data are presented as mean ± SD. Statistical significance versus the model group is indicated as *p < 0.05, **p < 0.01, and ***p < 0.001, ****p < 0.0001.

During the oral administration of solanesol to 8 ∼ 13-week-old db/db mice, both the metformin and solanesol groups exhibited a downward trend in body weight (Fig. 1A). White adipose tissue mass decreased significantly in the metformin group (White fat coefficient: 83.1 %, P < 0.05) and the medium- and high-dose solanesol group (White fat coefficient: 81.4 % and 82.7 %, P < 0.05) (Fig. 1B). Brown adipose tissue was significantly increased in the model group and significantly decreased in each treatment group of metformin and solanesol (Brown adipose tissue: 64.4 %, P < 0.001; 66.3 %, 69.3 % and 57.9 %, P < 0.01) (Fig. 1C). The db/db mice manifested classic leptin resistance phenotypes, exhibiting hyperphagia 38.6 g/day (1.8-fold of control group, p < 0.0001) and polydipsia 65 mL/day (2.6-fold of control group, p < 0.0001). Metformin treatment restored these aberrations by reducing food intake to 76.8 % of model levels (p < 0.0001) and water consumption to 54.7 % (p < 0.0001). Intriguingly, solanesol displayed non-canonical pathways beyond leptin signaling: medium-dose intervention selectively suppressed feeding behavior (84.1 % of model group, p < 0.01) without affecting water intake (Fig. 1D-E).

Longitudinal metabolic assessments revealed progressive deterioration of glucose homeostasis in db/db mice. At week 3, model group exhibited a 3.1-fold elevation in GTT AUC versus controls (p < 0.0001), which further escalated to 3.0-fold by week 5 (p < 0.0001). Therapeutic interventions demonstrated time-dependent efficacy: metformin and high-dose solanesol significantly reduced GTT AUC to 77.6 % and 78.7 % of model levels respectively at week 5 (both p < 0.05; Fig. 1F-G). Insulin resistance manifested earlier, with model mice showing 3.8-fold higher ITT AUC than controls at week 4 (p < 0.0001), persisting at 3.5-fold by week. Both metformin and medium-dose solanesol exhibited sustained insulin-sensitizing effects, reducing ITT AUC to 67.3 %/70.2 % of model levels at week 4 (p < 0.001), and further improving to 66.3 %/60.9 % by week 6 (p < 0.001; Fig. 1H-I). Notably, solanesol displayed differential dose**–**response patterns: glucose tolerance improved dose-dependently (high-dose efficacy at week 5), while insulin sensitivity peaked with medium-dose intervention (60.9 % reduction vs model at week 6), suggesting distinct mechanism prioritization −β-cell function enhancement versus peripheral insulin signaling optimization. Weekly measurements of fasting blood glucose levels over the first four weeks revealed a significant downward trend in the treatment groups (Fig. 1J). Post 6-week intervention, comprehensive glycemic profiling revealed significant metabolic improvements (Fig. 1K-M). The model group exhibited characteristic hyperglycemia markers: glycated serum protein (GSP: 227.0 ± 8.3 μ mol/L vs 174.5 ± 20.3 in controls, p < 0.001), hyperinsulinemia (INS: 34.0 ± 0.4 m IU/L vs 26.2 ± 0.5, p = 0.003), and elevated HOMA-IR (0.30 ± 0.01 vs 0.20 ± 0.01, p < 0.001). Solanesol demonstrated dose-independent glucose-lowering efficacy, with all dosage groups achieving GSP reductions comparable to metformin (74.8**–**78.4 % of model levels, p < 0.001). Notably, while insulin levels remained unchanged across groups (p > 0.05), HOMA-IR significantly decreased in both metformin (81.8 % of model, p < 0.01) and solanesol-treated mice (84.8 % across doses, p < 0.01), indicating improved insulin sensitivity without β-cell overstimulation- a distinct advantage over sulfonylureas in avoiding hypoglycemia risk.

The db/db mice developed profound dyslipidemia, exhibiting 2.2-fold, 1.6-fold, 1.6-fold, 3.3-fold, and 1.3-fold elevated total cholesterol (TC), triglycerides (TG), non-esterified fatty acids (NEFA), 3.3-fold increased LDL-C and HDL-C (all p < 0.01) compared to controls. High-dose solanesol outperformed metformin in lipid-lowering efficacy, reducing LDL-C to 34.6 % (vs metformin's 62.3 %, p < 0.0001) and TG to 50.6 % (vs 69.0 %, p < 0.01) of model levels, while dose-dependently normalizing TC (56.9**–**65.3 % of model), suggesting multi-targeted lipid homeostasis restoration of solanesol distinct from conventional therapies (Fig. 1N-R). These observation of improved dietary behavior and biochemical profiles suggests that solanesol have a positive modulatory effect on T2DM-related metabolic disturbances.

### Solanesol attenuates hepatic steatosis and dysfunction

**Fig. 2:**
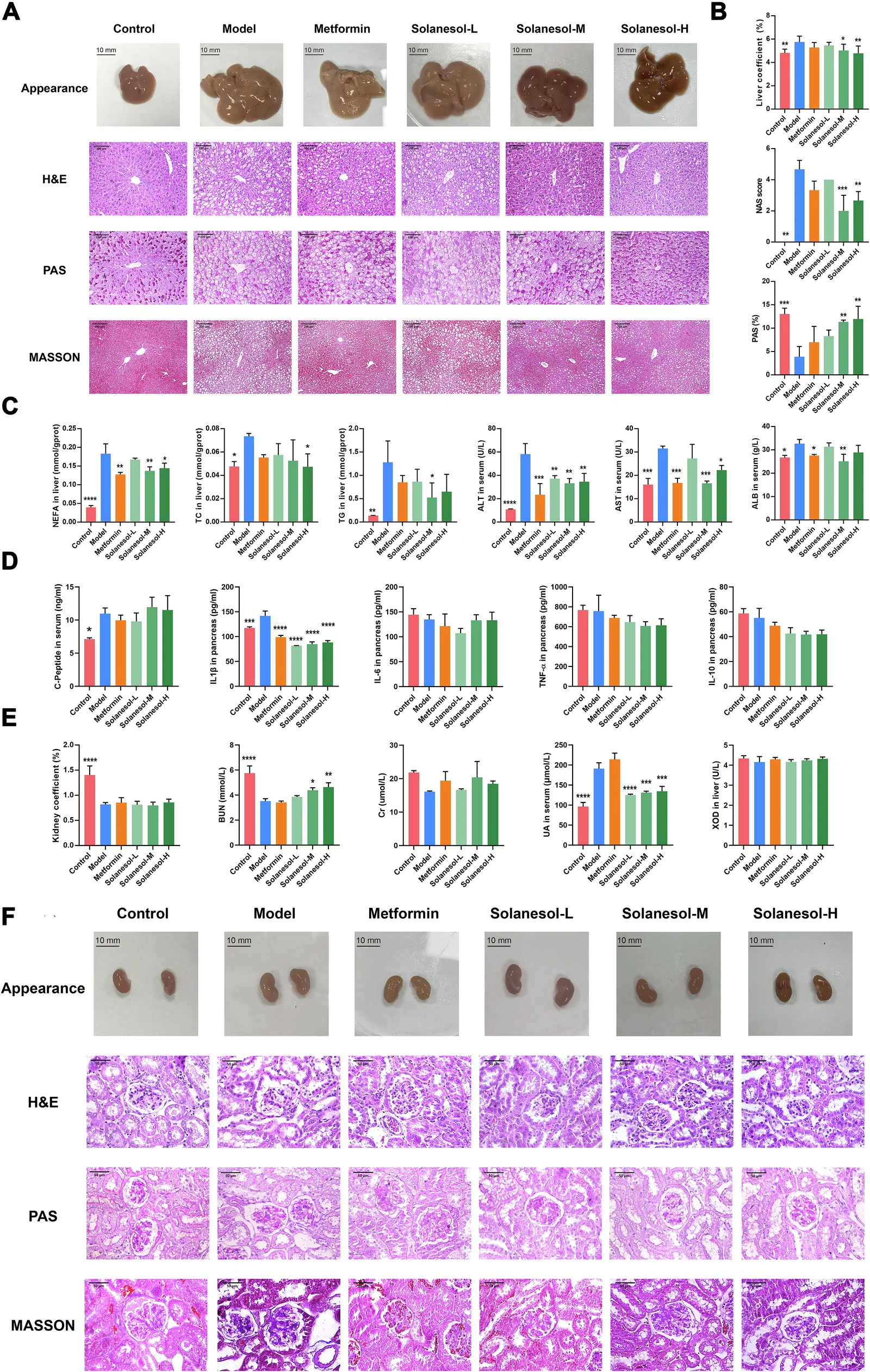
Solanesol ameliorates hepatic function, pancreatic inflammation and renal injury in T2DM mice. (A) Representative liver tissue sections stained with H&E (hematoxylin & eosin), PAS ((Periodic Acid-Schiff)), and Masson’s trichrome. (B) Liver index (liver-to-body weight ratio), histopathological scores of H&E and PAS staining. (C) Hepatic lipid content (NEFA, TC, TG), Serum levels of hepatic enzymes (ALT, AST) and albumin (ALB). (D) Serum C-peptide levels and pancreatic inflammatory cytokines (IL-1β, IL-6, TNF-α, and IL-10). (E) Kidney coefficient and serum renal function markers (BUN, Cr, UA), XOD activity of liver. (F) Renal histopathology (H&E, PAS, and Masson’s trichrome staining). Data are presented as mean ± SD. Statistical significance versus the model group is indicated as *p < 0.05, **p < 0.01, and ***p < 0.001, ****p < 0.0001.

Both gross morphology −showing enlarged, yellowish-pink livers with thickened edges and a significantly elevated hepatosomatic index (5.8 % vs 4.8 %, p < 0.01) revealed severe hepatic steatosis in model mice. Solanesol treatment at medium- and high-dose displayed near-normal hepatic architecture and reduced the liver index to 87.3 % and 83.1 % of the model levels, respectively (p < 0.05; Fig. 2A, B). Histologically, H&E staining confirmed pronounced macrovesicular steatosis in model mice with disrupted lobular structure, which was ameliorated by both metformin and solanesol intervention. PAS staining demonstrated solanesol's unique capacity to restore hepatic glycogen storage, with medium- and high-dose groups showing 2.9- and 3.1-fold increases in PAS-positive areas versus model (11.3 % and 12.1 % vs 3.9 %, p < 0.01), outperforming metformin's non-significant effect (Fig. 2B). Masson staining confirmed the absence of fibrosis across all groups, supporting the hepatoprotective profile and lack of toxicity of solanesol even at high concentrations.

Lipid profiling **(**Fig. 2C**)** revealed profound hepatic lipid dysregulation in model mice: NEFA (4.5-fold↑), TC (1.4-fold), and TG (9.1-fold↑, all p < 0.05). Medium-dose solanesol preferentially reduced TG by 59.4 %, while high-dose treatment lowered TC to 71.4 % of model level. Both solanesol doses and metformin comparably attenuated NEFA accumulation (77.8 % and 72.2 % of model, respectively, p < 0.05). These findings systematically indicate solanesol's multi-target hepatoprotective effects against T2DM-associated steatosis through lipid homeostasis restoration and metabolic reprogramming.

The db/db mice exhibited marked liver dysfunction, with serum ALT/AST levels elevated 5.3-fold and 2.0-fold versus controls (p < 0.001), accompanied by 1.2-fold hypoalbuminemia ALB (p < 0.05). Medium-dose solanesol demonstrated superior hepatoprotection, normalizing ALT to 57.1 % of model levels and AST to 52.7, correspond to metformin's effects (ALT: 40.2 %, AST: 53.2 %). Intriguingly, solanesol specifically ameliorated albumin synthesis (medium-dose ALB 77.0 % of model, p < 0.001; metformin ALB **84.4 %** of model, p < 0.05) **(**Fig. 2C**)**. The differential effects on transaminases versus albumin may suggest distinct mechanisms that could potentially involve both anti-inflammatory (e.g., NF-κB inhibition) [28], [29] and anabolic (e.g., mTORC1 activation) [30], [31] pathways, which merits further investigation into its potential therapeutic implications.

### Solanesol confers pancreatic and renal protection in diabetes

The model group exhibited a 1.5-fold elevation in fasting C-peptide levels versus controls (p < 0.05), consistent with compensatory hyperinsulinemia characteristic of insulin resistance. Notably, neither solanesol nor metformin interventions significantly altered circulating C-peptide (p > 0.74 vs model) or insulin concentrations (p > 0.49 vs model, Fig. 1L), as evidenced by stable β-cell secretory profiles across treatment groups (Fig. 2D). This dual pharmacological evidence**—**preserved pancreatic insulin output coupled with improved glycemic control (GSP: 227.0 ± 8.3 μ mol/L vs 174.5 ± 20.3 in controls, p < 0.001, Fig. 1K) **—**conclusively demonstrates that solanesol, like metformin, exerts its antidiabetic effects primarily through insulin sensitization rather than β-cell stimulation. Pancreatic cytokine profiling revealed a striking suppression of IL-1β by solanesol, with low, medium, high doses reducing this key mediator by 42.3 %, 40.0, and 37.6 %, respectively (all p < 0.0001), outperforming metformin's 30.2 % reduction. In contrast, TNF-α, IL-6, and IL-10 levels remained unaltered, pinpointing IL-1β as the specific therapeutic node, and suggesting protection occurs via an IL-1β-dependent anti-inflammatory pathway (Fig. 2D).

The db/db mice exhibited early metabolic nephropathy characterized by reduced renal index (0.8 % vs 1.4 % in controls, Δ = -42.9 %, p < 0.0001) and suppressed blood urea nitrogen (blood urea nitrogen [BUN]: 3.5 ± 0.2 vs 5.7 ± 0.58 mmol/L, p < 0.0001). Medium/high-dose solanesol unexpectedly elevated BUN to 120–130 % of model levels (p < 0.05), suggesting compensatory activation of hepatic ureagenesis. There was no difference in creatinine (Cr) among the groups. Crucially, solanesol uniquely attenuated hyperuricemia (serum uric acid: 65.4–70.5 % of model, p < 0.001) without XOD modulation in liver. These data suggest that its mechanism may involve the reprogramming of renal urate transporters (Fig. 2E). Histopathological examination of kidney sections indicated only minor lesions, such as mild segmental and focal sclerosis in the model group (PAS staining), with no signs of inflammation, fibrosis, or typical diabetic nephropathy. No significant pathological changes were observed in the kidneys of metformin and solanesol-treated mice (Fig. 2F), These findings thus suggest a renal protective effect of solanesol even at low concentrations, while also indicating an absence of nephrotoxicity even at the higher administered dose in our experiment.​.

### Solanesol restructures gut microbiota composition

**Fig. 3:**
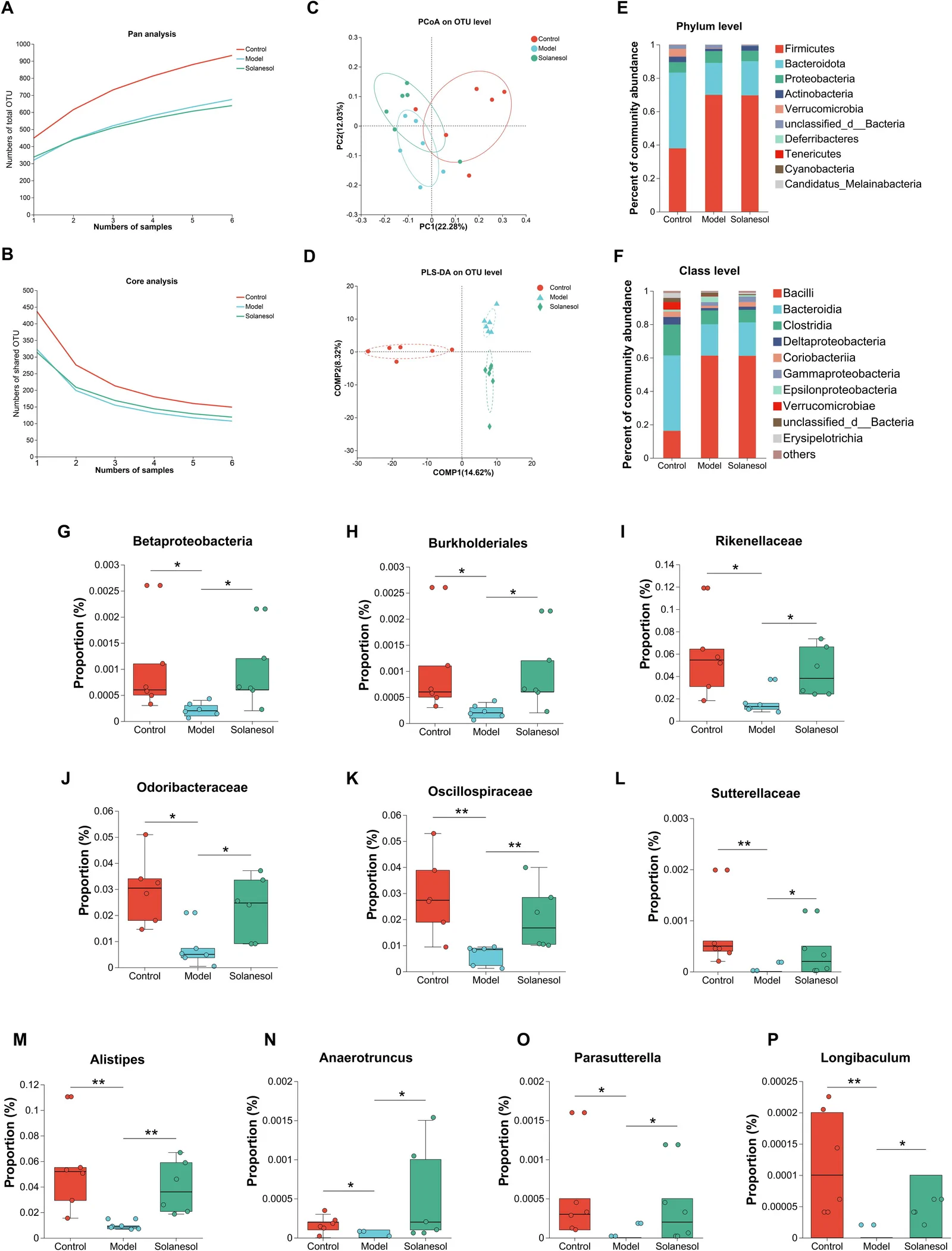
Solanesol modulates fecal microbiota composition in db/db mice. (A) Pan-microbiome OTU (Operational Taxonomic Unit) analysis. (B) Core microbiome OTU analysis. (C) PCoA (Principal Coordinates Analysis) at OTU level. (D) PLS-DA (Partial Least Squares Discriminant Analysis) at OTU level. (E) Phylum-level taxonomic distribution of gut microbiota. (F) Class-level taxonomic distribution. (G) Relative abundance of β-Proteobacteria at class level. (H) Differential microbiota at order level. (I-L) Differential microbiota at family level. (M−P) Differential microbiota at genus level. All data were analyzed and visualized using the Majorbio I-Sanger Cloud Platform (www.i-sanger.com).

A total of 1.48 million high-quality sequences were obtained, with an average of 82,420 ± 16,294 sequences per sample. Microbial sequencing data were clustered into operational taxonomic units (OTUs). The Pan-OTU and Core-OTU curves demonstrated microbial community stability within groups as sample size increased (Fig. 3A-B). Principal coordinates analysis (PCoA) and partial least squares discriminant analysis (PLS-DA) revealed significant differences in fecal microbiome composition among the control, model, and mid-dose solanesol groups (P = 0.002) (Fig. 3C-D).

Bacterial community structure differed across taxonomic levels among groups. The microbiota was predominantly composed of Firmicutes and Bacteroidetes at the phylum level (>83 % total), with increases in Firmicutes and decreases in Bacteroidetes observed in both model and solanesol groups compared to controls (Fig. 3E). Similar shifts were seen at the class level, where Bacilli increased while Bacteroidia and Clostridia decreased in these groups (Fig. 3F). Notably, β-Proteobacteria (class) and Burkholderiales (order) were more abundant in control and solanesol groups than in the model group (Fig. 3G, H). At finer taxonomic resolutions, families such as Rikenellaceae, Odoribacteraceae, Oscillospiraceae, and Sutterellaceae (Fig. 3I-L), as well as genera including *Alistipes*, *Anaerotruncus*, *Parasutterella*, and *Longibaculum* (Fig. 3M−P), were consistently enriched in control and solanesol-treated mice. The increased abundance of these beneficial bacterial groups suggests an improvement in gut health in T2DM mice.

### Solanesol established a new balance of intestinal metabolites

**Fig. 4:**
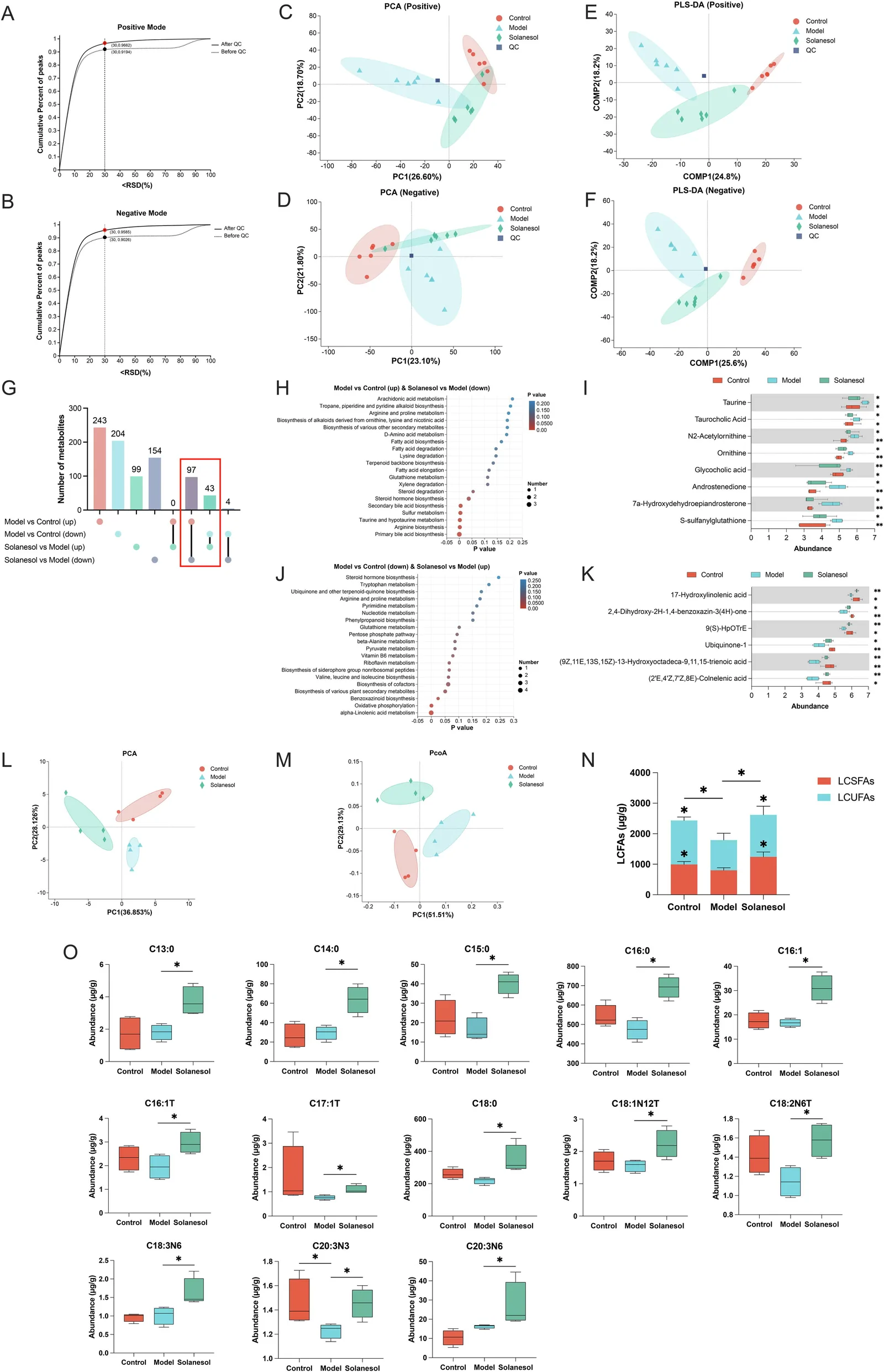
Solanesol restructures the intestinal metabolome in db/db mice. (A-B) Relative standard deviation (RSD) assessment of metabolite data stability in positive (A) and negative (B) ion modes (dashed line: pre-processing; solid line: post-processing; Raw data shown as single solid line). (C-D) Principal component analysis (PCA) of metabolites detected in positive (C) and negative (D) ion modes. (E-F) Partial least squares-discriminant analysis (PLS-DA) of intra- and inter-group metabolite separation. (G) Statistical analysis of differential metabolites. (H) KEGG pathway enrichment of 97 solanesol-downregulated differential metabolites. (I) Significantly downregulated metabolite markers affected by solanesol. (J) KEGG pathway enrichment of 43 solanesol-upregulated differential metabolites. (K) Significantly upregulated metabolite markers affected by solanesol. (L-M) PCA (L) and PCoA (M) analyses of long-chain fatty acid (LCFA) detection data. (N) Abundance analysis of long-chain fatty acids (LCFAs), long-chain saturated fatty acids (LCSFAs), and long-chain unsaturated fatty acids (LCUFAs). (O) Quantitative detection of LCFAs. All data were analyzed and visualized using the Majorbio I-Sanger Cloud Platform (www.i-sanger.com).

Fecal metabolomic profiling identified 562 and 912 metabolites in positive and negative ion modes, respectively. Quality control assessment revealed excellent data reliability, with relative standard deviation (RSD) values < 0.3 and cumulative peak proportions > 70 % (Fig. 4A-B). PCA demonstrated clear segregation among the control, model, and solanesol groups (Fig. 4C-D). This separation was further confirmed by PLS-DA (VIP > 1.0, p < 0.05 in Wilcoxon test), indicating significant intergroup metabolic differences (Fig. 4E-F).

Comparative analysis identified 243 upregulated and 204 downregulated metabolites in the model group verse controls. Whereas, 99 metabolites were upregulated and 154 were downregulated in the mid-dose solanesol group relative to the model group (Fig. 4G). Metabolite set enrichment analysis of the 97 model-upregulated metabolites (consistently low in control and solanesol groups) identified significant involvement in primary and secondary bile acid biosynthesis, arginine biosynthesis, taurine and hypotaurine metabolism, sulfur metabolism, and steroid hormone biosynthesis (Fig. 4H). Key altered metabolites in these pathways included Taurine, Taurocholic Acid, N2-Acetylornithine, Ornithine, Glycocholic acid, Androstenedione, 7a-Hydroxydehydroepiandrosterone, and S-sulfanylglutathione (Fig. 4I). Conversely, the 43 model-downregulated metabolites (restored by solanesol) were enriched in α-linolenic acid metabolism, oxidative phosphorylation, and benzoxazinoid biosynthesis (Fig. 4J), with notable changes in metabolites included 17-Hydroxylinolenic acid, 2,4-Dihydroxy-2H-1,4-benzoxazin-3(4H)-one, 9(S)-HpOTrE, Ubiquinone-1, (9Z,11E,13S,15Z)-13-Hydroxyoctadeca-9,11,15-trienoic acid, and (2′E,4′Z,7′Z,8E)-Colnelenic acid (Fig. 4K).

Fecal metabolomics revealed distinct long-chain fatty acid (LCFA) profiles among the control, model, and mid-dose solanesol groups, as confirmed by PCA and PCoA (Fig. 4L-M). Total LCFA content was significantly lower in the model group (1791.86 μg/g) than in the control (2434.00 μg/g) and solanesol (2617.25 μg/g) groups, corresponding to increases of 35.84 % and 46.06 %, respectively. These shifts were reflected in both long-chain saturated (LCSFAs) and unsaturated fatty acids (LCUFAs): Compared to the model group, LCSFA levels were 23.74 % higher in controls and 54.95 % higher in the solanesol group, while LCUFA levels increased by 45.62 % and 38.88 %, respectively (Fig. 4N). Notably, solanesol administration significantly elevated the abundances of multiple saturated species-including tridecanoate (C13:0), myristate (C14:0), pentadecanoate (C15:0), palmitate (C16:0), and stearate (C18:0), as well as unsaturated forms such as palmitoleate (C16:1), palmitelaidate (C16:1t), 10-*trans*-heptadecenoate (C17:1T), elaidate (C18:1N12T), linolelaidate (C18:2N6T), γ-linolenate (C18:3N6), 11–14-17-eicosatrienoate (C20:3N3), and homo-γ-linolenate (C20:3N6) (Fig. 4O). Further analysis revealed an interesting pattern: short-chain fatty acids (SCFAs, C2-C5) were decreased in the model group and showed a further reduction after solanesol treatment (Supplemental Fig. 1A). This may suggest enhanced intestinal absorption of SCFAs, resulting in lower fecal excretion [32], [33]. In contrast, medium-chain fatty acids (MCFAs, C6-C10) were elevated in model mice but trended toward normalization following solanesol intervention (Supplemental Fig. 1B). Together with broader metabolomic data, these results indicate that solanesol exerts a distinctive regulatory influence over intestinal fatty acid metabolism, ultimately reestablishing microbial-host metabolic balance and supporting systemic protection through gut-organ axes.

### Integrated multi-omic profiling of the murine hepatic transcriptome and proteome

**Fig. 5:**
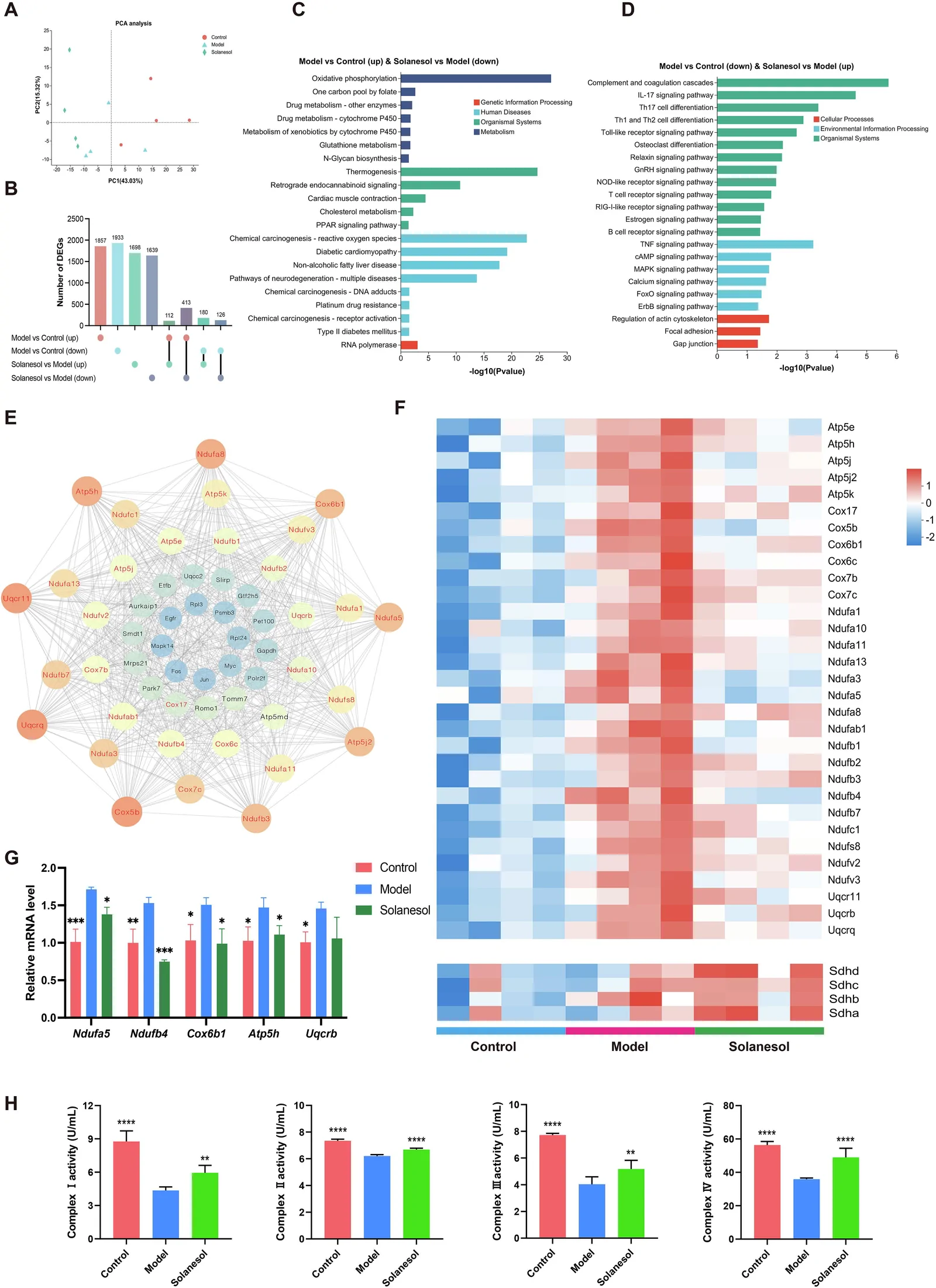
Hepatic transcriptomic profiling and validation of solanesol-mediated metabolic regulation. (A) Principal component analysis (PCA) demonstrating robust intergroup segregation among control, diabetic model, and solanesol-treated cohorts (n = 4/group). (B) Quantifying differentially expressed genes (DEGs) (|log2FC|>1, FDR < 0.05). (C-D) KEGG pathway enrichment of intersecting DEGs: C: Model-up & solanesol-down genes；D: Model-down & solanesol-up genes. Color gradients indicate pathway classifications. (E) Protein-protein interaction (PPI) network of core DEGs (53 nodes with degree ≥ 20), highlighting mitochondrial respiratory chain hubs. (F) Heatmap of OXPHOS-related DEGs showing overexpression in model (Complexes I/III/IV/V) and normalization by solanesol, excluding Complex II (Sdha-d). (G) qRT-PCR validation of representative OXPHOS genes corroborating transcriptomic trends. (H) ELISA demonstrates solanesol's restoration of mitochondrial complex activities. Data are presented as mean ± SD. Statistical significance versus the model group is indicated as *p < 0.05, **p < 0.01, and ***p < 0.001, ****p < 0.0001.

High-depth RNA sequencing of hepatic tissues from the control, model, and mid-dose solanesol groups generated 82.63 Gb of high-quality data (6.13 Gb/sample, Q30 ≥ 94.61 %), meeting stringent thresholds for downstream analyses. PCA showed clear segregation (Fig. 5A), with differential expression analysis revealing 1,857 upregulated and 1,639 downregulated genes in the diabetic model versus controls. This dysregulation was markedly modulated by solanesol treatment, which resulted in 1,933 upregulated and 1,698 downregulated DEGs compared to the model group (Fig. 5B). Functional annotation revealed that solanesol-downregulated DEGs were enriched in metabolic pathways (e.g., oxidative phosphorylation [OXPHOS]; cholesterol metabolism; PPAR signaling) and disease-associated processes (e.g., NAFLD, T2DM, diabetic cardiomyopathy) (Fig. 5C). Upregulated DEGs were predominantly associated with immune-inflammatory pathways, including B/T-cell receptor signaling, Th-cell differentiation, and cytokine cascades (e.g., IL-17, Toll-like receptor, NOD/RIG-I-like receptors, TNF, MAPK, FoxO) (Fig. 5D). Protein-protein interaction (PPI) network analysis of 593 core DEGs using Cytoscape identified 53 high-confidence hub nodes (degree ≥ 20) (Fig. 5E), with top-ranked genes (Cox5b, Uqcrq, Uqcr11, Atp5h, Ndufa8, Cox6b1, Ndufa5, Atp5j2 and Ndufb3) predominantly associated with mitochondrial respiratory chain complexes I, III, IV, and V. Notably, 31 OXPHOS-related DEGs exhibited marked overexpression in diabetic models but were normalized by solanesol treatment. This regulatory specificity was absent in complex II (Sdha-d) (Fig. 5F). qRT-PCR validation of representative genes (Ndufa5, Ndufb4, Cox6b1, Atp5h, Uqcrb) confirmed the transcriptomic trends (Fig. 5G). However, the observed upregulation of these DEGs may reflect an ineffective compensatory or stress response, as ELISA assays indicated reduced enzymatic activity in the model group (Fig. 5H). This impairment in electron transport chain function likely contributes to diminished ATP synthesis and elevated ROS production, further aggravating insulin resistance and liver injury **−** a self-perpetuating pathological cycle. In contrast, solanesol administration effectively reversed these alterations, demonstrating its ability to restore mitochondrial integrity and disrupt this detrimental feedback loop. These findings suggest that the amelioration of mitochondrial dysfunction by solanesol may involve a sophisticated regulatory network, potentially encompassing the stability of complex subunits, their assembly efficiency, post-translational modifications, and cofactor availability [34], [35].

**Fig. 6:**
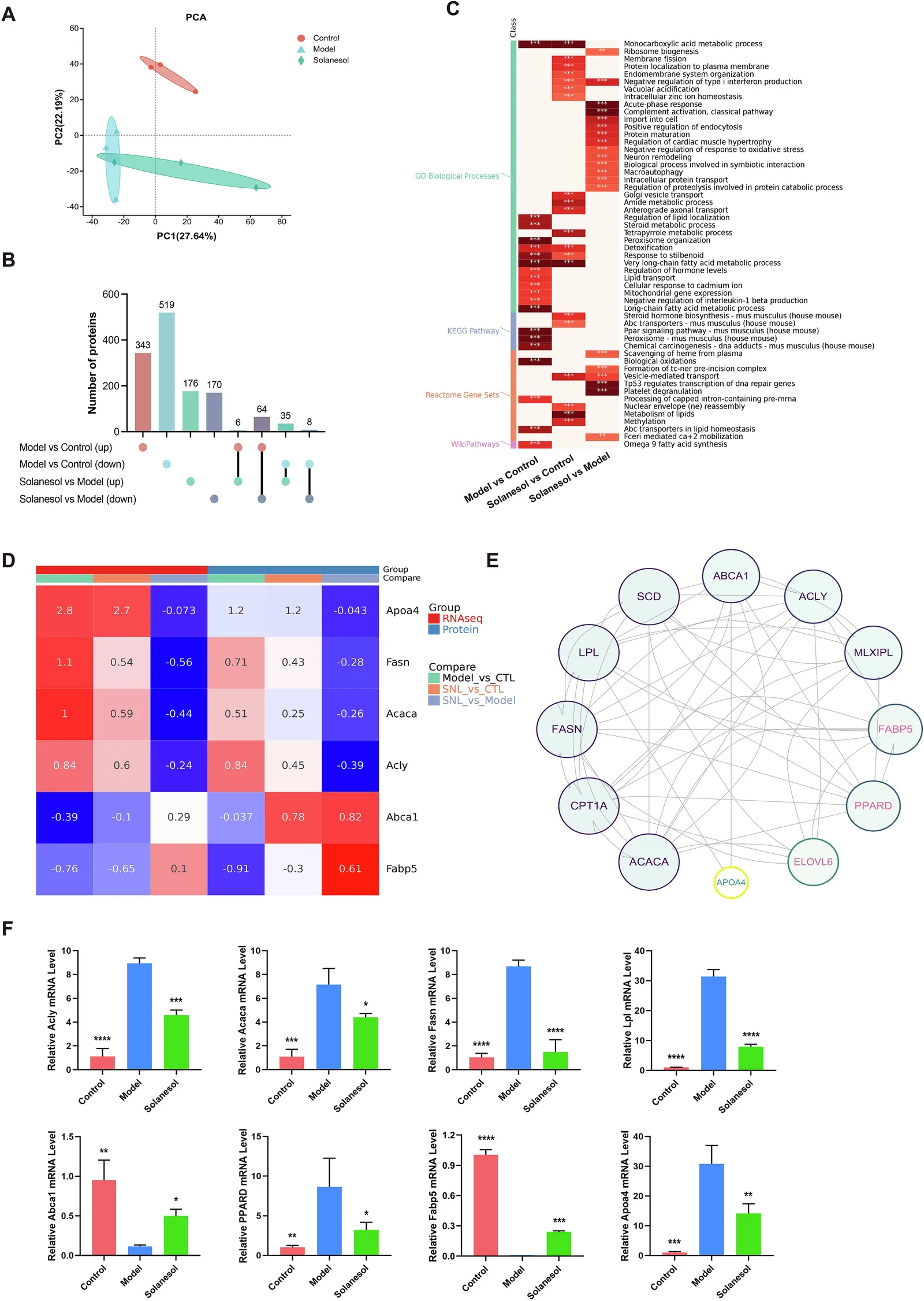
Integrated transcriptome (n = 4/group) and proteome profiling (n = 3/group). (A) Principal component analysis (PCA) demonstrating robust intergroup segregation among control, diabetic model, and solanesol-treated cohorts. (B) Hepatic proteomic different expressed proteins (|log2FC|>1, p value < 0.05). (C) Functional enrichment of DEPs. (D) Consensus lipid regulators (Acly, Acaca, Fasn, Apoa4, Abca1, Fabp5, PPARδ, Lpl) showing concordant expression trends across transcriptomic and proteomic platforms. (E) PPI network of solanesol-targeted lipid nodes. (F) qRT-PCR validation of representative solanesol-targeted lipid node corroborating transcriptomic trends. Data are presented as mean ± SD. Statistical significance versus the model group is indicated as *p < 0.05, **p < 0.01, and ***p < 0.001, ****p < 0.0001.

Proteomic analysis revealed significant intergroup separation by PCA (Fig. 6A). Global analysis identified 5,972 quantifiable proteins, with 217 and 242 differentially up- and down-regulated expressed proteins (DEPs) in mid-dose solanesol-treated vs. model groups, respectively (p adj < 0.05, |log2FC| > 1.5) (Fig. 6B). Functional annotation revealed significant enrichment in two core processes: lipid metabolic pathways (including lipid localization, steroid and long-chain fatty acid metabolism, and transport) and oxidative stress responses (encompassing peroxisome organization, mitochondrial gene expression, PPAR signaling, and biological oxidations) (Fig. 6C). Multi-omics integration of transcriptomic and proteomic datasets identified six core regulators (Apoa4, Acaca, Acly, Fasn, Abca1, Fabp5), in terms of lipid metabolism, transport, localization, with consistent expression trends across all platforms (Fig. 6D). PPI network analysis delineated a central lipid regulatory axis involving Abca1, Apoa4, and Fabp5, interconnected with Acly-Acaca (Fig. 6E). Subsequent qPCR analysis of eight network-derived genes demonstrated that solanesol concurrently suppressed de novo lipogenesis (DNL) genes (Acly, Acaca, Fasn) and enhanced lipid-binding/transport genes (Fabp5, Abca1), the latter being associated with a subsequent downregulation of apolipoprotein gene Apoa4. Collectively, the altered expression of these genes contributed to reduced ​​free fatty acids (FFA)​​ levels. This decrease consequently reversed the high expression of ​​PPARD​​ observed in the model group, ultimately mitigating hepatic inflammation (Fig. 6F).

## Discussion

This study established solanesol as a phytochemical with anti-diabetic efficacy at remarkably low doses (1 ∼ 15 mg/kg/day) in db/db mice. Following a 6-week intervention, solanesol elicited metabolic improvements comparable to those achieved with high-dose metformin (200 mg/kg/day). Importantly, at a dosage of 5 mg/kg/day, solanesol showed superior efficacy in ameliorating hyperglycemia (assessed by ITT), hyperlipidemia (reflected in white adipose tissue, TC, and LDL levels), hyperuricemia (UA), hepatic steatosis (TG content, NAS score, AST, and ALB levels), and pancreatic inflammation (IL-1β −dependent). Furthermore, solanesol preserved β-cell mass without stimulating insulin secretion, highlighting its unique non-secretagogue profile. ​​Notably, solanesol conferred concurrent anti-hyperuricemia activity (ΔUA = -44.6 % vs. model) in the T2DM context, suggesting potential in addressing a clinical challenge relevant to the 17 %–22 % of diabetic patients with comorbid gout [36], outperforming metformin's neutral (ΔUA=+8.2 %) or potentially detrimental uric acid profile. Its XOD-independent mechanism may circumvent hepatotoxicity risks with conventional urate-lowering drugs. Moreover, its favorable renal safety profile positions solanesol as a promising candidate for managing diabetes with hyperuricemia, particularly where renal protection is crucial. These outcomes were achieved at 1/40 the clinical equivalent metformin dose, highlighting the compound's considerable pharmacological potency and safety advantage.

Integrative transcriptomic-proteomic analyses delineated solanesol’s hepatic protection mechanism: (i) suppression of de novo lipogenesis through Acly/Acaca/Fasn inhibition; (ii) enhancement of lipid clearance via Fabp5-driven β-oxidation and Abca1-mediated cholesterol efflux; (iii) mitochondrial optimization by modulating electron respiratory chain complexes Ⅰ, Ⅱ, Ⅲ, IV, V and normalizing OXPHOS activity; (iv) attenuation of inflammation through Lpl-Pparδ inhibition. Emerging evidence positions hepatic mitochondrial OXPHOS as a pivotal therapeutic target in early-stage diabetes management. Across multiple diabetic models—including streptozotocin-induced, db/db, and high-fat diet-fed mice-hepatic Complex I/II-dependent OXPHOS activity exhibits a biphasic trajectory: marked elevation during early disease progression followed by late-phase decline [37]. Unlike metformin's singular inhibition of Complex I [38], solanesol exerts a coordinated modulation patten of both the activities and expression levels of electron transport chain complexes (I, Ⅱ, III, IV, and V; e.g., Ndufa5, Ndufb4, Cox6b1, Atp5h, Uqcrb), thereby effectively rebalancing pathological OXPHOS disturbance to improve mitochondrial dysfunction. This multi-complex intervention achieves superior mitochondrial redox balance at 1/40 that of metformin, while circumventing compensatory metabolic adaptations commonly observed with single-target agents. It suggests that solanesol has a unique capacity to restore bioenergetic homeostasis without requiring structural modifications or advanced delivery systems − a critical advantage for clinical translation.

The present study confirms the Acly-Acaca-Fasn pathway as a key mechanism underlying solanesol's amelioration of hepatic steatosis in T2DM [39], [40]. Acly, a pivotal enzyme catalyzing citrate conversion to acetyl-CoA and oxaloacetate, drives hepatic de novo lipogenesis and cholesterol synthesis. Solanesol administration significantly suppressed Acly expression in diabetic mice, thereby attenuating hepatic steatosis and ballooning degeneration. This inhibition extended to acetyl-CoA carboxylase (Acaca)-the rate-limiting enzyme for malonyl-CoA production-and Fasn, effectively blocking the Acly-Acaca-Fasn lipogenic cascade. Notably, Acly suppression correlated with reduced hepatic stellate cell activation and fibrotic marker, highlighting solanesol's anti-fibrotic potential [39], [40]. Concomitantly, solanesol upregulated Abca1, promoting cholesterol efflux and suppressed VLDL assemble. Beyond lipid regulation, Abca1 activation triggered JAK2-STAT3 signaling in macrophages, inhibiting NF-κB-driven cytokine release. This dual metabolic-immune modulation was also observed in pancreatic islets and skeletal muscle, positioning solanesol as a novel Abca1 agonist for systemic metabolic regulation [41], [42]. Furthermore, solanesol uniquely upregulated Fabp5, a lipid chaperone facilitating mitochondrial/peroxisomal β-oxidation. This enhancement promoted fatty acid flux toward energy production, thereby reducing lipotoxicity and improving insulin sensitivity. Fecal metabolomics corroborated this mechanism, showing elevated long-chain fatty acid, suggesting Fabp5-mediated lipid mobilization [43]. We propose that the coordinated upregulation of Abca1 and Fabp5 reduces intracellular lipid content, subsequently diminishing the cellular demand for Apoa4 and Lpl. This collective reduction in free fatty acids alleviates hepatocyte lipid stress, leading to downregulation of abnormally elevated PPARδ. These mechanistic changes may represent one potential pathway contributing to the attenuation of hepatic inflammation, or alternatively, may constitute secondary responses following the amelioration of hepatic steatosis. Collectively, these findings establish that solanesol intervention induces a novel gene expression profile conducive to metabolic homeostasis in fatty liver disease.

A key and intriguing finding of this study is that solanesol exerts multi-layered and sophisticated regulatory effects on the gut ecosystem and its systemic impacts. Firstly, solanesol intervention significantly remodeled the gut microbiota, characterized by the enrichment of beneficial taxa including key SCFA producers *Alistipes* and *Anaerotruncus* [44], as well as *Odoribacteraceae* and *Parasutterella*, which are associated with bile acid metabolism and PPAR signaling activation [45], [46]. This microbial shift was accompanied by a comprehensive recalibration of host fatty acid metabolism. Fatty acid quantification revealed a chain-length-dependent regulatory pattern for solanesol: on one hand, it further reduced the levels of SCFAs in feces (C2-C5, Supplemental Fig. 1A), potentially indicating enhanced intestinal absorption and systemic utilization of these beneficial fatty acids [32], [33]; on the other hand, it decreased MCFAs and normalized the levels of LCFAs (Fig. 4O), suggesting that solanesol not only enhances the bioactivity of SCFAs but also corrects the diabetic dysregulation of MCFAs and LCFAs, thereby suppressing harmful lipid accumulation and ameliorating the inflammatory milieu [47]. This gut-centric regulation holds significant systemic implications. For instance, via the gut-liver axis, enhanced SCFA absorption may improve gut barrier function and reduce systemic inflammation, thereby alleviating hepatic inflammatory stress and insulin resistance − effects that likely underlie solanesol's marked hepatoprotective and glucose-lowering actions. Furthermore, involvement of the gut-brain axis is suggested by the elevated levels of anti-inflammatory long-chain unsaturated fatty acids, particularly within the α-linolenic acid pathway (palmitoleate [C16:1], γ-linolenate [C18:3N6], and eicosatrienoate [C20:3N]). These lipidomic shifts, especially the increase in γ-linolenate (C18:3N6), provide further evidence for solanesol's neuroprotective effects via enhanced brain-derived neurotrophic factor (BDNF) signaling [12], [13], [14], [15], positioning solanesol as a potential paradigm-shifting therapeutic agent bridging metabolic and neurodegenerative disorders. In summary, solanesol does not merely correct individual metabolic parameters; it systematically restores overall homeostasis along the gut-liver-brain axis [48]. By simultaneously modulating microbial composition and the fatty acid profile across all chain lengths, it promotes a healthier gut microenvironment that translates into multi-organ protection. This broad-spectrum, systems-level action underscores solanesol's unique potential as a therapeutic strategy for T2DM and its comorbidities. Nevertheless, long-term studies are urgently needed to elucidate the dynamics of fatty acid fluxes, metabolic turnover, and response heterogeneity.

Beyond the BDNF-related pathway, solanesol's neuroprotective potential may also be mediated through enhanced mitochondrial cofactor biosynthesis. Notably, solanesol significantly elevated ubiquinone-1 (CoQ1) levels in T2DM mice; CoQ1 is a mitochondrial electron transport chain mediator and precursor to CoQ10. This aligns with its demonstrated neuroprotective efficacy in bipolar disorder models, where solanesol activated Sirt-1 signaling, restored mitochondrial membrane potential, and reduced neuronal apoptosis [15]. Crucially, solanesol achieved these effects at low doses (5 mg/kg/day) −40-fold lower than metformin (200 mg/kg/day) in mice. Conventional CoQ10 supplementation exhibits critical pharmacological shortcomings. Its extreme hydrophobicity results in lower oral bioavailability (≤5%) [49], [50], [51], necessitating paradoxically fail to improve glycemic control or lipid profiles in diabetic patients, as evidenced by systematic review and *meta*-analysis of 7 trials (n = 356) [52]. These findings redefine solanesol from a CoQ10 synthesis intermediate [11] to a potent standalone therapeutic agent. Its capacity to bypass CoQ10′s pharmacokinetic limitations while eliciting broader metabolic-neurological benefits suggests a novel “precursor-to-therapeutic” repositioning strategy. Solanesol is expected to be a cost-effective alternative to high-dose CoQ10 supplements. Future studies will compare solanesol’s efficacy against synthetic CoQ10 in diabetic neuropathy models and elucidate its role in modulating CoQ enzyme cascades.

While this study establishes solanesol's early-phase therapeutic efficacy in T2DM model over 8 ∼ 13 weeks, the relatively short intervention period and modest sample size (n = 6 per group) **—**though consistent with common practices in preclinical studies and sufficient to detect significant treatment effects-may have limited statistical power for capturing more subtle trends such as the body weight reduction observed with metformin [53]. Future studies employing larger sample sizes, diverse disease models, and extended intervention periods (≥6 months) will be essential to validate solanesol's chronic protective potential against diabetic complications (e.g., retinopathy, nephropathy, neuropathy) and strengthen the generalizability of these findings. Mechanistically, deeper interrogation of tissue-specific signaling networks is warranted, including spatiotemporal expression profiling of mitochondrial respiratory chain complexes in hepatic tissue, single-cell resolution analysis of β-cell-immune crosstalk, and spatial transcriptomics of renal urate transporter niches. Furthermore, clinical translation will require additional steps such as large-cohort evaluations, systematic safety assessments, and long-term efficacy studies. Nevertheless, the observed multi-organ protection-reflected by reductions in hepatic steatosis (↓59 %), pancreatic IL-1β (↓42 %), and renal uric acid (↓45 %) −along with intestinal metabolic reprogramming capacity, suggests that solanesol may represent a novel natural compound with the potential to simultaneously address multiple metabolic disturbances in T2DM, offering a promising alternative to conventional single-target therapies.

## Conclusion

Integrated multi-omics analyses reveal solanesol as a multi-target phytotherapeutic agent that coordinately ameliorates T2DM pathophysiology through: (1) Hepatic metabolic reprogramming via Acly/Acaca/Fasn-mediated lipogenesis suppression, Fabp5/Abca1/Apoa4/Lpl-driven β-oxidation, cholesterol efflux and PPARδ inhibition; Mitochondrial optimization of electron transport chain complexes I, Ⅱ, III, IV, and V. (2) Pancreatic IL-1β inflammasome inhibition without β-cell overstimulation; (3) Renal urate transporter remodeling and inflammation inhibition; (4) Gut microbiota restructuring with α-linolenic acid metabolism lipid enrichment. Systems pharmacology validates its superior network proximity to metabolic syndrome targets vs metformin (5 mg/kg/day vs 200 mg/kg/day. i.g.), positioning it as a first-in-class natural modulator of the “metabolic quartet” − dysglycemia, dyslipidemia, hyperuricemia, and multi-organ inflammation.

## Ethics statement

This animal experiment has been approved by the Guangzhou Miles Animal Ethical and Welfare Committee (IACUC-MIS20230035) according to the Guide for the Care and Use of Laboratory Animals of the National Institutes of Health.

## CRediT authorship contribution statement

**Wenji Zhang:** Conceptualization, Data curation, Formal analysis, Investigation, Methodology, Project administration, Resources, Supervision, Validation, Visualization, Writing – original draft, Writing – review & editing. **Wenli Cheng:** Writing – original draft, Validation, Software, Investigation. **Jiaqi Fu:** Data curation, Investigation, Methodology. **Ruidong Zeng:** Data curation, Methodology. **Zhangying Wang:** Conceptualization, Supervision, Project administration, Resources. **Jingbo Zhou:** Software, Supervision. **Yibo Chen:** Conceptualization, Supervision, Data curation, Funding acquisition. **Wenjuan Zhang:** Conceptualization, Data curation, Formal analysis, Funding acquisition, Investigation, Methodology, Project administration, Resources, Software, Supervision, Validation, Visualization.

## Declaration of competing interest

The authors declare that they have no known competing financial interests or personal relationships that could have appeared to influence the work reported in this paper.

## Figures and Tables

**Supplementary Figure 1:**